# DrunkChain: Blockchain-Based IoT System for Preventing Drunk Driving-Related Traffic Accidents

**DOI:** 10.3390/s23125388

**Published:** 2023-06-07

**Authors:** Hamza Farooq, Ayesha Altaf, Faiza Iqbal, Juan Castanedo Galán, Daniel Gavilanes Aray, Imran Ashraf

**Affiliations:** 1 Department of Computer Science, University of Engineering & Technology, (UET), Lahore 54890, Pakistan; m.hamza.arif786@gmail.com (H.F.); faiza.iqbal@uet.edu.pk (F.I.); 2 Higher Polytechnic School, Universidad Europea del Atlántico, Isabel Torres 21, 39011 Santander, Spain; juan.castanedo@uneatlantico.es (J.C.G.); daniel.gavilanes@uneatlantico.es (D.G.A.); 3 Department of Projects, Universidade Internacional do Cuanza, Cuito EN250, Bié, Angola; 4 Research Group on Foods, Nutritional Biochemistry and Health, Fundación Universitaria Internacional de Colombia, Bogotá 111311, Colombia; 5 Universidad Internacional Iberoamericana Campeche, Campeche 24560, Mexico; 6 Universidad Internacional Iberoamericana Arecibo, Puerto Rico, PR 00613, USA; 7 Department of Information and Communication Engineering, Yeungnam University, Gyeongsan 38541, Republic of Korea

**Keywords:** Internet of Things, blockchain, drunk driver detection, network security, MQ3

## Abstract

Traffic accidents present significant risks to human life, leading to a high number of fatalities and injuries. According to the World Health Organization’s 2022 worldwide status report on road safety, there were 27,582 deaths linked to traffic-related events, including 4448 fatalities at the collision scenes. Drunk driving is one of the leading causes contributing to the rising count of deadly accidents. Current methods to assess driver alcohol consumption are vulnerable to network risks, such as data corruption, identity theft, and man-in-the-middle attacks. In addition, these systems are subject to security restrictions that have been largely overlooked in earlier research focused on driver information. This study intends to develop a platform that combines the Internet of Things (IoT) with blockchain technology in order to address these concerns and improve the security of user data. In this work, we present a device- and blockchain-based dashboard solution for a centralized police monitoring account. The equipment is responsible for determining the driver’s impairment level by monitoring the driver’s blood alcohol concentration (BAC) and the stability of the vehicle. At predetermined times, integrated blockchain transactions are executed, transmitting data straight to the central police account. This eliminates the need for a central server, ensuring the immutability of data and the existence of blockchain transactions that are independent of any central authority. Our system delivers scalability, compatibility, and faster execution times by adopting this approach. Through comparative research, we have identified a significant increase in the need for security measures in relevant scenarios, highlighting the importance of our suggested model.

## 1. Introduction

Road accidents have emerged as a significant problem and can result in both human casualties and property loss. With more speeding automobiles on the road, the likelihood of automobile collisions has risen. Approximately 80 million automobiles are manufactured worldwide, and the number continues to rise [1]. Since 1991, only 39 highways, motorways, expressways, and vital routes have been constructed, which is fewer than the number of automobiles generated annually. As a result, there are significantly more automobiles on the road, and even minor carelessness might put road users at risk. Pakistan has stringent rules on alcohol consumption [2]; however, as one of the most economically developing nations, it is observed that the lowest-class citizens, such as laborers, consume the most alcohol [3]. Numerous Pakistani commercial drivers are addicted to narcotics and alcohol, which increases the number of deadly accidents.

Currently, the government and traffic police in Pakistan do not use new technology in the traffic system. According to the WHO’s (World Health Organization’s) worldwide status report on road safety [4], in 2010, there were 4448 deaths at the accident scenes, and an additional 27,582 deaths resulting from road accidents. According to one report, intoxicated drivers were involved in 150 fatal road accidents in Pakistan over a period of ten months [5]. Pakistan has a national rule against drinking and driving, but its implementation is only at a rate of 30%. The enforcement of laws against driving under the influence of alcohol is carried out by the authorities [2]. If a driver is tired or intoxicated, the appropriate police officials will utilize roadblocks and inspect each vehicle individually. They must evaluate drivers based on indicators such as the smell of alcohol on the driver’s breath, the driver’s physical appearance, and driving performance. Fuel officers visually assess the suspect and the suspect’s car in order to determine the suspect’s BAC. Despite best practices, police officers cannot determine the alcohol concentration of every driver by analyzing the driving behavior. This method is inadequate since it is sometimes inaccurate and prone to bias and human error. Implementing more automated and sensor-driven technology can reduce the time and government resources required to detect drunk driving. Table 1 displays the distribution of road-related fatalities in Pakistan.

This research presents a system that utilizes IoT (Internet of Things) technology and enhanced security measures to prevent drunk driving accidents. The transmission of information is secured, and authentication and permission mechanisms have been introduced alongside IoT devices. Using blockchain to encrypt data is a step forward in the search for viable solutions, and there is a high likelihood of its official implementation. The main contributions of this study are as follows:

- We propose a novel technique for detecting driver intoxication by monitoring both the alcohol concentration in the driver’s breath and the quality of the vehicle’s operation. As a result, even if the alcohol sensor is intentionally covered, an intoxicated driver cannot remain undetected.
- We propose a compact, easy-to-install hardware module that uploads the BAC, motion, and GPS data of the driver at regular intervals to the blockchain. Every driver with the installed module on their vehicle will have a recorded data history that depicts the driver’s trust profile.
- We highlight the immutability, scalability, and security features that make the platform more trustworthy and reliable. It is not possible to change the transaction history, regardless of the level of authority; hence, the guilty will eventually be penalized.
- We also elaborate on possible attacks, such as data mutability, identity theft, and man-in-the-middle attacks, as well as how they can be prevented.

The remainder of the manuscript is arranged as follows: Section 2 sheds light on existing work on alcohol detection and prevention. Section 3 describes the methodology and workflow of how the system operates, the hardware and software components and modules that have been used thus far, and their intended uses. Section 4 provides a description of the attacks covered by the system, along with their brief explanations and graphical representations. In addition, a comparison of the proposed system is carried out with the existing systems. Finally, this study is concluded in Section 6.

## 2. Literature Review

Due to the limited time available each day for activities such as work, chores, and responsibilities, it is common for individuals to drive quickly in order to complete tasks. Frequent road accidents are caused by individuals who move quickly and disregard traffic regulations. It has been observed that the majority of drivers disregard traffic regulations. Most public attempts to avoid traffic wardens occur if a traffic light is malfunctioning or when there is additional personnel on the road managing heavy traffic [6]. Table 2 provides a summary of the distinct findings from various research studies in this field, along with a discussion of the focal points and gaps identified in the literature. The categories listed in the table are as follows:

- 3 If comprehensive work is done, well-explained, and tested.
- 2 If work is somewhat conducted and explained.
- 1 If a tad bit of an idea is given.
- 0 If work is not explained at all.

**Figure 1:**
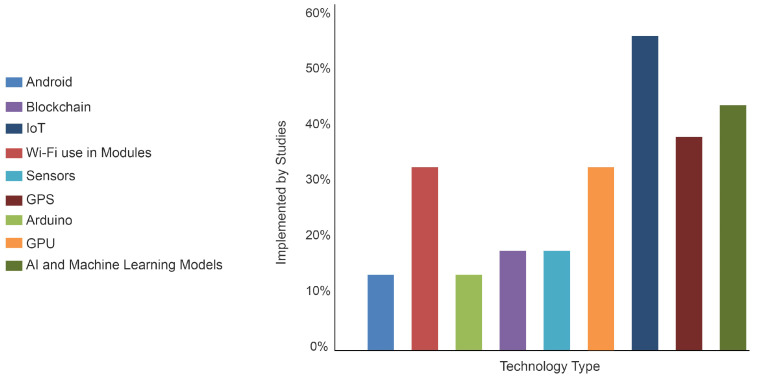
Technology distribution of each system presented in existing studies.

For better visual comprehension, the research articles are categorized based on the underlying technology used, as depicted in Figure 1. Further details can be found in Table 3. Considering the work in [16], it is a good idea to protect the system from cyberattacks. However, IoT devices typically lack a solid foundation when it comes to security and privacy. Interoperability is another crucial issue to consider. This is not handled very well by IoT devices. The system should be sufficiently able to reduce the need for micromanaging security issues. This solution combines blockchain technology with IoT devices that serve as security capsules for internet communication. Decentralized authentication will also be implemented in this domain. Modern vehicles with IoT-enabled devices are used to monitor traffic scenarios based on the behaviors and locations of drivers. Through the cloud, different traffic and road conditions are continuously forecasted. The dataset is then classified according to a variety of circumstances using real-time modules of machine learning. Here, blockchain and IoT devices are implemented, resulting in the creation of a secure system [17].

The authors of [23] proposed a system that predicts if the vehicle’s operator is alert or drowsy. This is accomplished by integrating IoT devices and video streaming processors. In conjunction with the vehicle’s positioning, the eye aspect ratio is analyzed, and in the event of a collision, a notification is sent to the police station and the nearest emergency assistance. The face detection algorithm is used to achieve precise results for eye blinking and eye positioning. This system could be enhanced by incorporating an alcohol detection sensor into the module. The MQ-3 sensor improves the process of apprehending an intoxicated driver by detecting BAC on the driver’s breath. By integrating a Q3 sensor and an Arduino Uno microcontroller interface with a GPS (global positioning system) system that monitors the driver and detects any errant motions in the vehicle’s motion, the system can detect any erratic driving behavior. This hybrid approach categorizes a driver’s data based on the driver’s level of intoxication. The ITP (information transfer process) flow is complex in the government sector. This must be streamlined and swiftly action-oriented. To improve processing efficiency, blockchain technology is implemented. There is a flow that controls and distributes the stages of the entire process. This flow restructures the business and operational phases and supports the e-government infrastructure [20]. Studies in the domain of trust-based IoT networks can be integrated with vehicular networks. In [26], the authors identified and highlighted existing solutions and trust models of IoT. Their survey identified existing challenges, issues, and future directions. Similarly, another study [27] presented trust-based solutions for IoT in smart buildings. Another study [28] utilized trust models of IoT to present security solutions for smart city networks. In [29], the authors provided a context-based trust model to provide edge intelligence and security to IoT nodes. In [30], the authors highlighted blockchain architecture, applied domains, and platforms. They provided a detailed description of the existing security threats of blockchain technology. An important survey [31] was conducted in the domain, which provided detailed guidelines for driving safety with sensing in vehicular communications. The authors utilized artificial intelligence-based collision avoidance mechanisms and a testbed-based analysis to present solutions for collision avoidance.

In [18], the authors proposed using a smartphone to determine whether or not a person is intoxicated. The application is installed on a mobile device in the same manner as any other app. The application is then subject to the behavior and position of the vehicle when it is in motion. If the system detects any behavior indicating that a person is intoxicated, it alerts the concerned party to seize or rescue the individual. This alert can be sent to the local police station or a family member. Several IoT devices are integrated and intercommunicated to enable smart homes; this is the future of technology. On the other hand, the likelihood of cyberattacks is high, and the repercussions can be much more severe. The system presented in [21] utilizes blockchain to protect against cyberattacks. When edge computing is integrated with transaction protocols, such as smart contract blockchains, it prevents data privacy breaches. The reduction in the latency of IoT devices is an additional factor that should be considered. Scalability plays an important role in this type of system, especially when time is a critical factor for reporting the location, monitoring driver behavior, or generating alerts in sensitive situations, such as heavy traffic.

An AI (artificial intelligence) module was integrated with IoT devices in [25] to ensure that the drunk driver was constantly monitored and arrived at their destination safely. In the event of an emergency or in drunk driving, information regarding the driver’s speed and location is collected, analyzed, and reported as necessary. Similarly, in [19], the dangers and driving behaviors were evaluated. The system identifies situations where something goes wrong, which is referred to as the detection of sleepiness. When detected, the system immediately alerts the relevant parties when an accident is predicted. When one of the factors reaches an alarming rate, this solution detects the driving pattern. For this, it considers speed, unpredictable driving, and speed control. The system is installed on a smartphone with detectors and employs data correction and classification algorithms to filter the data. After the data are cleaned from noise and undergo a cross-correlation procedure, the speed is estimated, and the driver’s behavior is determined, based on the real-time data.

Along the same direction, the authors of [22] focused on reducing the causes of motor vehicle collisions. Using IoT devices and IR (infrared) sensors, the vehicle’s current location and velocity are determined. The RFID (radio frequency identification) system determines the number of vehicles in motion. A module is used for GSM (global system for mobile) communications, and a solution is created by combining the three modules, each of which employs one sensor.

From the above-discussed research works, it was found that road accidents can be significantly reduced by adopting new technologies. However, existing solutions are predominantly limited in the context of security, scalability, latency, or privacy. The use of IoT devices with a security perspective, such as blockchain, and advancements to increase efficiency, such as the use of machine learning algorithms, must be integrated in a manner that prevents the drawbacks associated with these solutions.

## 3. Methodology

This work aims to continuously monitor the BAC level and vehicle stability and to transfer data to the blockchain in real time. If the driver’s BAC level exceeds the threshold, the driver is considered intoxicated. The system is installed in the vehicle and is connected to the internet. The motion sensor and alcohol sensor are used in conjunction to determine whether or not the driver is intoxicated. Thus, if the driver employs a method to obstruct the alcohol detection sensor, the motion sensor remains operational and can be used to determine whether the driver is intoxicated. The system consists primarily of two modules: the IoT module and the blockchain module.

### 3.1. IoT Module

Using a variety of sensors, this module monitors the driver’s BAC level and generates a data stream for transmission to the blockchain module. The data stream includes the driver’s alcohol concentration, the vehicle’s motion stability, and its location. The IoT hardware module is comprised of several sensors and IoT devices. In the hardware description, the specific components utilized by this module are outlined. The circuit and flow diagrams are described in the hardware architecture section.

#### 3.1.1. Hardware Description

The hardware devices, including sensors that are used to sense and collect data from the vehicle, are the Arduino breakout board, MQ3 alcohol detector, MPU6050 accelerometer, gyroscope module, and NEO-6m GPS module.

**Figure 2:**
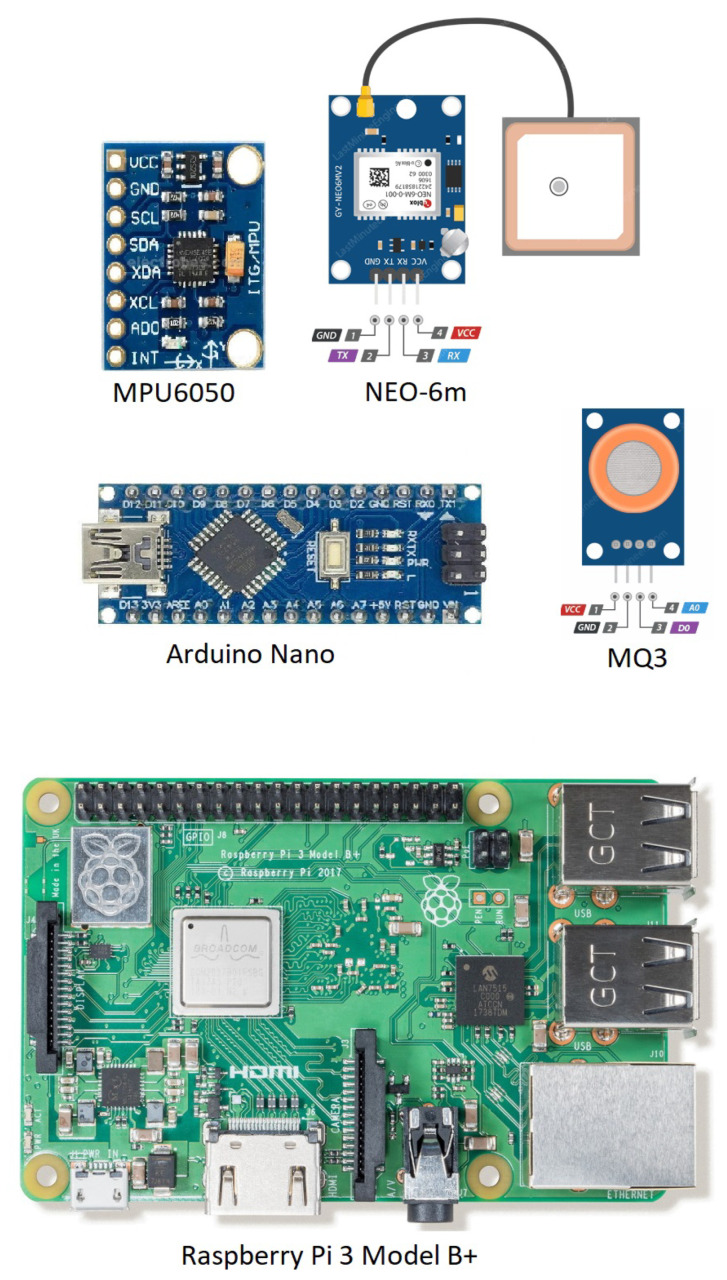
IoT devices that are used in the DrunkChain.

Arduino Nano is an Atmega328p-based small breakout board. It functions as a central board to which the sensors are attached. It collects data from sensors and prepares it for serial communication transmission. It has built-in analog-to-digital conversion so analog sensors can be connected directly. It has an integrated power supply module, allowing sensors and other devices connected to this board to be powered directly by this board. It can be powered by either a USB (universal serial bus) or an external power supply. The input voltage ranges between 7 and 12 V. Using the IDE (integrated development environment) made available by Arduino, the embedded C can be used to program Arduino. Figure 2 depicts the front and back views of the Arduino Nano breakout board, as well as a description of each pin.

The MQ3 sensor is the most important component in the alcohol detection process. In the realm of metal oxide semiconductors, this component is classified as a chemiresistor type. It can detect alcoholic beverages, ethanol, and cigarette smoke. While the driver is operating the vehicle, this sensor determines the driver’s BAC. Pin 2, also known as GND (ground), is connected to the ground, and pin 1, also known as VCC (voltage common collector), is connected to a power supply that delivers 5 V to it. It is capable of analog output and digital output. The analog output is handled by the fourth pin. The range of the analog output is anywhere from 0 to 5 V. The digital output is handled exclusively by Pin 3. The digital output has two predetermined values: 0 volts (meaning sober) and 5 volts (meaning drunk), with 0 volts meaning false and 5 volts meaning true. Figure 2 illustrates the sensor diagram with the mentioned pinout.

The sensor that monitors the vehicle’s motion stability is MPU6050. It is an IMU (inertial measurement unit) with six axes. It is equipped with a three-axis accelerometer and a three-axis gyroscope, enabling the system to measure rotation along all three axes, static acceleration due to gravity, and dynamic acceleration due to motion. The sensor consists of eight conductive pins. As the sensor is 5 V-rated, the VCC pin is connected to 5 V, and the GND pin is connected to 0 V. The SDA (serial data) and SCL (serial clock) pins are connected to Arduino’s A4 and A5 pins, respectively. I^2^C [32] is used to facilitate communication between the sensor and Arduino. The I2C protocol uses two data lines for data communication: a SCL that is pulsed at regular intervals by the Arduino controller board, and a SDA pin over which data are transmitted between the sensor and Arduino. The Arduino library [33] for mpu6050 includes the ’getMotionInterruptStatus’ function, which determines if the motion stability level is within the threshold value. The system was tested with multiple values, and 20 is determined to be the most suitable. If the vehicle accelerates or decelerates rapidly in any direction to the extent that its motion stability level exceeds the predetermined threshold, the vehicle will be deemed unstable and the driver will be committing careless driving. The sensor’s appearance is depicted in Figure 2, along with pin labels.

The NEO-6m GPS module’s primary purpose is to ascertain the precise geographic location of a moving vehicle at any given time. It is able to achieve a high-tracking sensitivity of −161 dB because it can pick up transmissions from as many as 22 satellites simultaneously, across a total of 50 separate channels. This makes it possible to achieve such a high-tracking sensitivity. In terms of the amount of power it consumes, it only needs 45 mA (milliamperes) of current. The GPS module can perform location updates at a rate of up to 5 times per second, and they are accurate to a horizontal distance of 2.5 m. Serial communication is the method through which it interacts with Arduino. This indicates that Arduino receives readable GPS data once every second from the device. The TTFF (time-to-first-fix) of the positioning engine used in the U-Blox 6 is less than one second, earning it a reputation for being extremely quick. The PSM (power save mode), which helps reduce power consumption by selectively switching off certain components, is one of its most notable features. This mode is responsible for turning on and off certain components of the receiver. The GPS module Neo 6 m is in charge of handling satellite positioning duties. The geolocation of the vehicle is what we are trying to determine with this GPS module’s help. The current latitude and longitude of the vehicle are communicated by it. The module’s low power consumption, ease of interface, and relatively high level of accuracy compared to other modules currently on the market are factors that led to its selection. The GPS module, along with the pin description and the antenna, can be seen in Figure 2.

#### 3.1.2. Hardware Architecture

**Figure 3:**
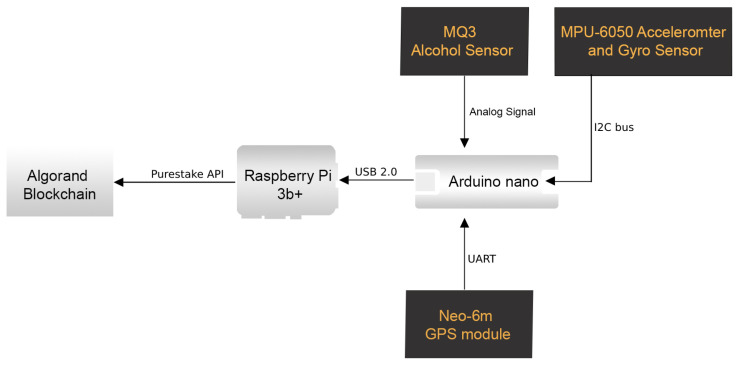
Block diagram of the DrunkChain device.

The IoT hardware module’s block diagram is shown in Figure 3. The Arduino board is linked to the Alcohol sensor, motion sensor, and GPS module. Arduino Nano collects and compiles sensor data as central units. The data are reported continuously via serial communication at 1 s intervals. The data have formats, such as alcohol. Value: <ALCOHOL SENSOR VALUE>, motion stability: <MPU6050 MOTION STABILITY VALUE>, latitude: <GPS Latitude>, longitude: <GPS LONGITUDE>}, where ALCOHOL SENSOR VALUE> ranges from 0 to 1023, and MPU6050 MOTION STABILITY VALUE> can be either 0 or 1, indicating whether the vehicle is stable or not, GPS Latitude> contains the geographical latitude of the vehicle, and GPS Longitude> contains the geographical longitude of the vehicle.

**Figure 4:**
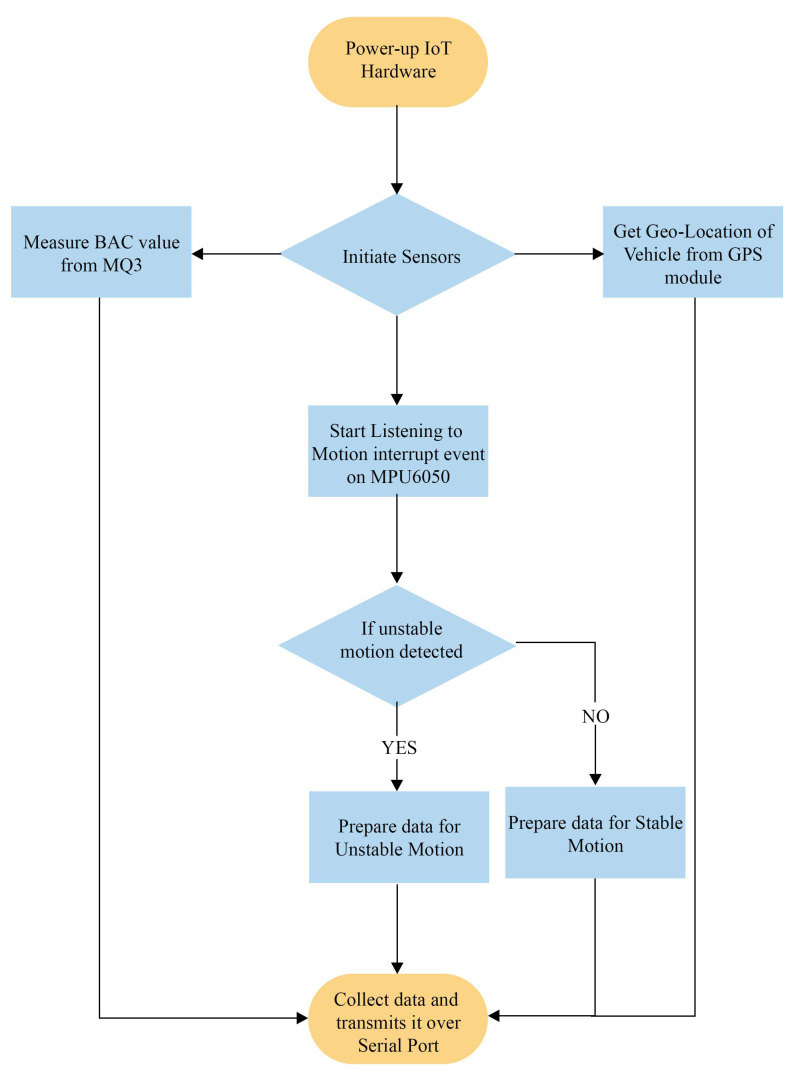
Flow diagram of the DrunkChain device.

Figure 4 illustrates the IoT hardware module’s workflow. Arduino begins collecting data from sensors as soon as the module is powered up. The MQ3 sensor is straightforward because it outputs the sensor’s analog value. The analog-to-digital converter within Arduino is used to read the signal. The MPU6050 motion sensor is connected to Arduino via the ’Itextsuperscript2C’ bus. Arduino begins to listen for the motion interrupt. When the motion instability threshold is exceeded, the event is triggered and the vehicle is deemed unstable. Arduino prepares data accordingly. Arduino is connected to the NEO-6m GPS module via serial communication. Every second, Arduino reads the latitude and longitude of the vehicle from this module. Arduino transmits the collected data via the serial port at a one-second interval.

### 3.2. Blockchain Module

Driver monitoring systems are insecure and susceptible to numerous cyberattacks, such as hacking and DDoS (distributed denial-of-service) attacks. Any user with access to the central database has the ability to modify the record at any time. Blockchain technology is considered a solution for these types of issues. The blockchain module is comprised of two sub-modules that are elaborated on in the subsequent sections.

#### 3.2.1. Module Architecture

**Figure 5:**
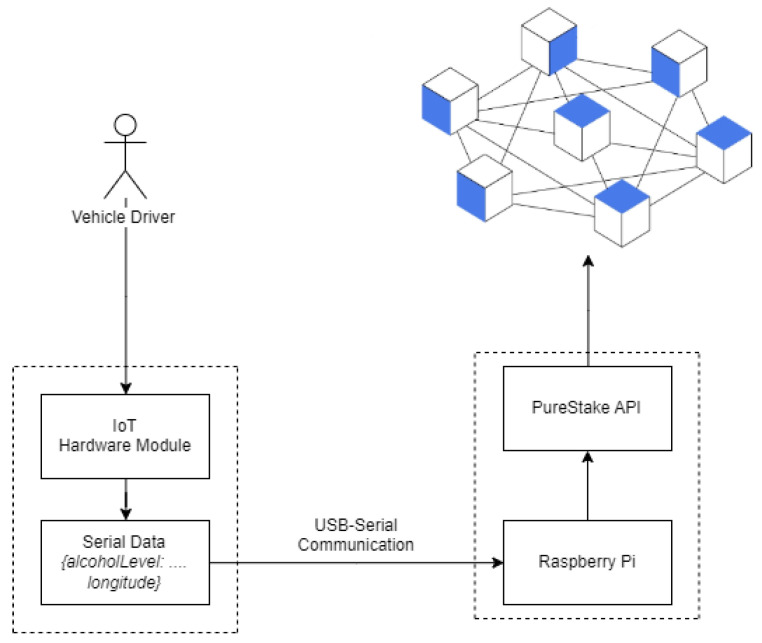
The overall system architecture of DrunkChain.

The IoT hardware module detects and transmits the vehicle’s BAC and motion stability via serial communication. This module is connected via USB to Raspberry Pi in order to receive the data via USB-serial communication. In the system, Raspberry Pi 3 Model B+ is utilized. In the current implementation, Raspberry Pi is a single-board computer that runs Ubuntu 20.04 [34]. Ubuntu is a Linux-based operating system designed for a variety of purposes. The application is written using Node.js. The [35] Algorand blockchain is utilized by the system. Algorand is significantly more efficient in terms of time, which makes it suitable for IoT implementation. The PureStake API (application programming interface) service simplifies the onboard procedure for the Algorand network. Utilizing PureStake’s existing infrastructure platform provides developers with easy access to Algorand’s native REST APIs. Raspberry Pi continues to perform blockchain transactions at 10 s intervals indefinitely. The driver’s BAC level and motion data are added to the blockchain every 10 s. Once data are written, it is written permanently, and it is technically impossible to modify the transaction details. Figure 5 depicts the system’s overall architecture diagram. The computer utilized by the system is a Raspberry Pi 3 Model B+. This compact single-board computer has numerous interfaces, making it suitable for IoT projects.

**Figure 6:**
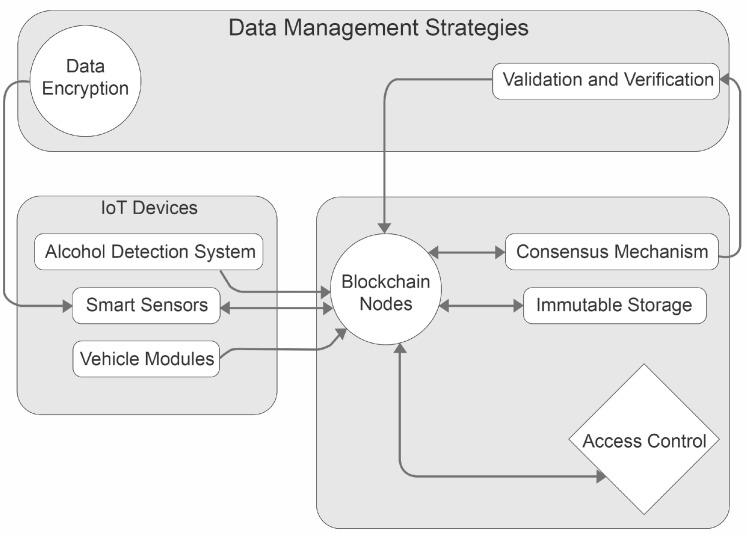
DrunkChain comprehensive architecture showing the interaction between IoT devices and blockchain modules.

Figure 6 shows a comprehensive architectural diagram showing data management strategies, IoT devices, and blockchain network interaction of DrunkChain. Smart sensors, alcohol detection systems, and vehicle modules are examples of IoT devices that are represented by the “IoT Devices” subgraph. Blockchain nodes, the consensus mechanism, immutable storage, and access control comprise the blockchain network. These are represented by the “Blockchain Network” subgraph. Data encryption and validation/verification are two data management strategies that are represented by the “Data Management Strategies” subgraph. The connections between the diagram’s elements are indicated by arrows, which indicate the movement of data and operations. For example, IoT devices are linked to blockchain nodes, which represent the data collection process. The consensus mechanism, immutable storage, and access control are linked to the blockchain nodes, demonstrating the flow of information and processes inside the blockchain network.

**Figure 7:**
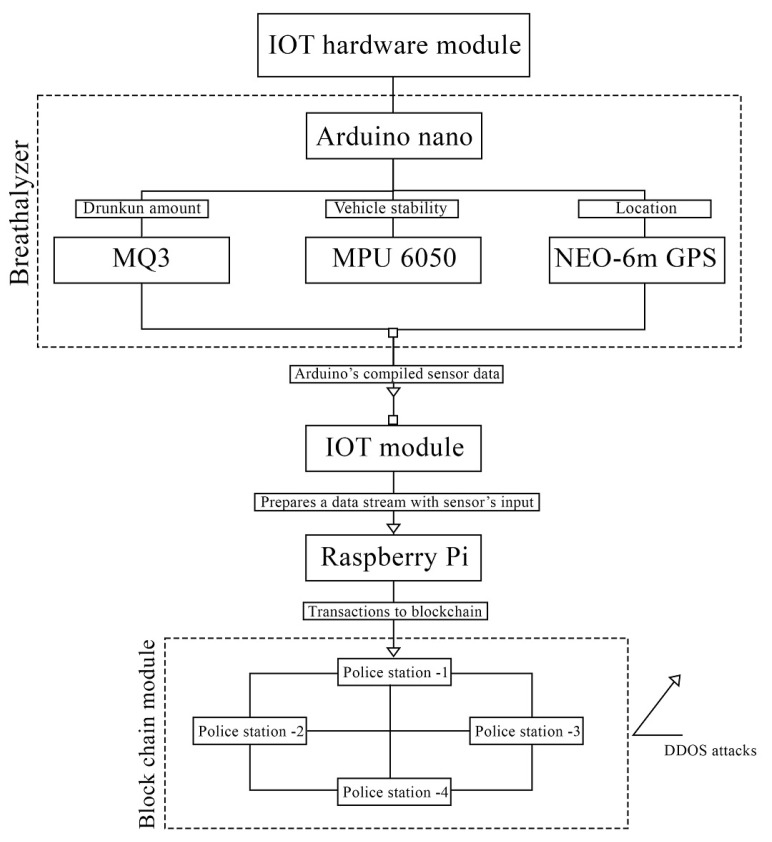
DrunkChain system/network model.

A system model consisting of various IoT modules that are networked with each other is displayed in Figure 7. The schema is set up so that the Arduino Nano, which is the one that is connected to the three sensors (the MQ3, the MPU 6050, and the NEO-6m GPS), is the one that gathers data from the sensors and sends them to Raspberry Pi in the form of serial communication. Raspberry Pi then computes the data and sends them to the blockchain.

**Figure 8:**
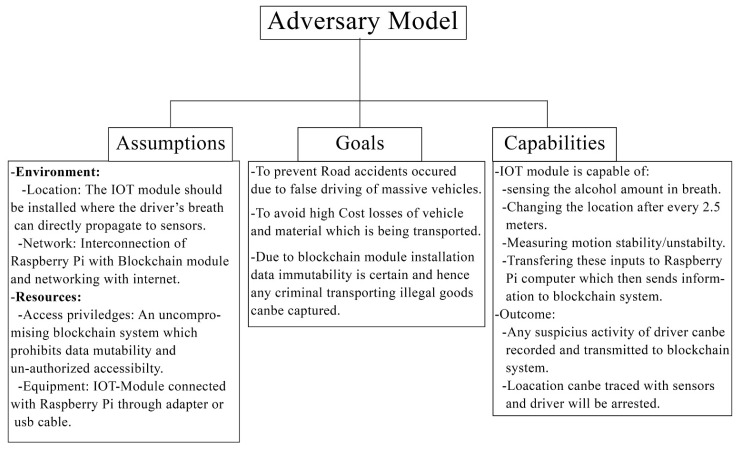
DrunkChain adversary model.

Figure 8 displays the adversary model that has been discussed. In this model, we delve into the details of the physical location where the module is placed, the network sources to which the IoT module will be continuously connected for sending the driver’s BAC level to the blockchain, and the resources required for constructing the physical structure of the design. In addition, the problem statement and requirements for the project must be specified. Through this process, the design’s necessary capabilities are refined.

**Figure 9:**
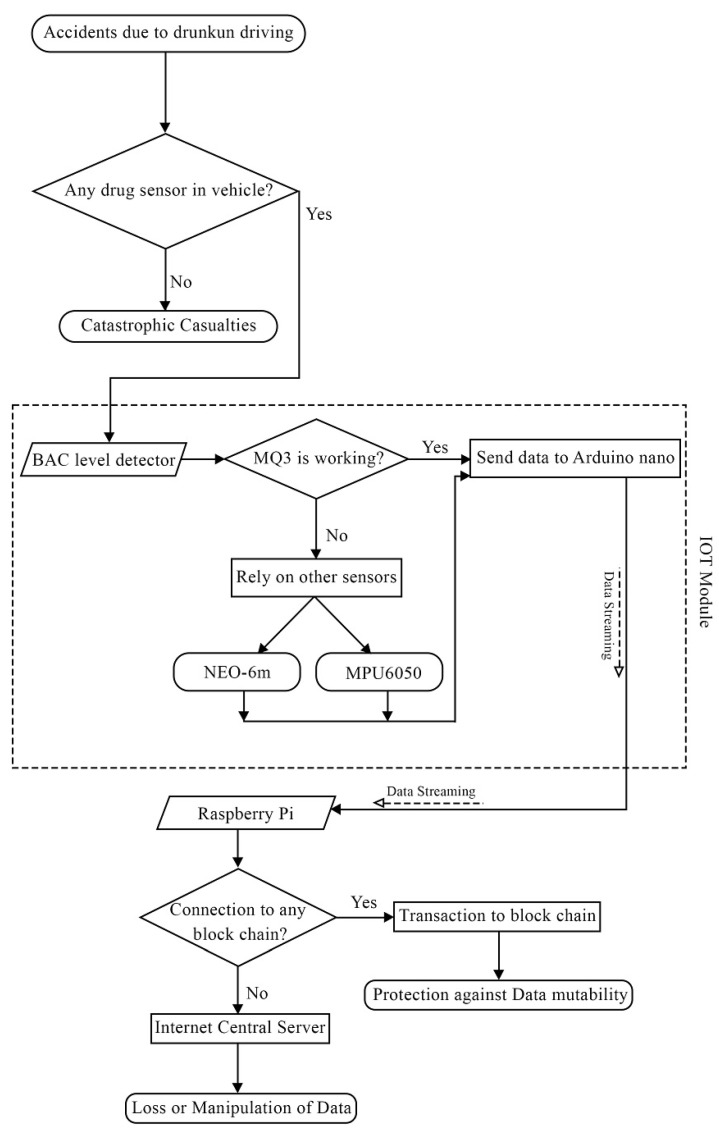
Diagrammatic representation of the proposed design.

If the MQ3 alcohol detector is unable to detect the BAC level due to the driver’s mouth direction, the MPU 6050 and NEO-6m GPS will trace the motion stability and location of the vehicle. After this, a data stream will be created to send the information over to the Raspberry Pi computer. This process is illustrated in detail in Figure 9. This information is stored on the blockchain by Raspberry Pi.

**Figure 10:**
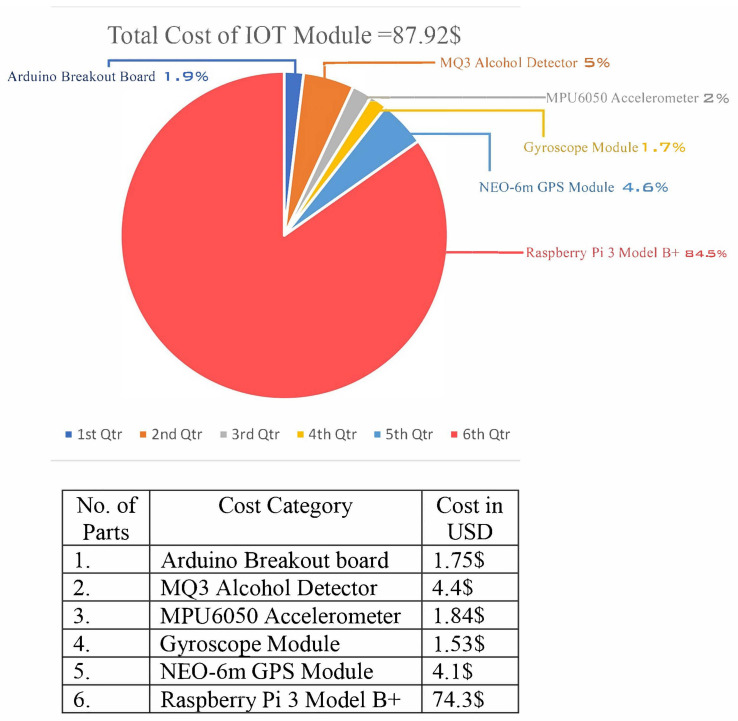
Cost analysis of the DrunkChain system.

The cost breakdown of the design modules is shown in Figure 10; its purpose is to ensure that the desired product will develop a financial boundary equal to a certain amount, in this case, USD 87.92 We are able to buy an alcohol sensor, a location tracker, a vehicle stability sensor, a gyroscope module, an Arduino breakout board, and Raspberry Pi with this amount of money.

#### 3.2.2. Challenges Catered to by Blockchain

- Enhanced overall security of the system: The IoT modules are low-powered and computationally inferior devices. Modern security and cryptography algorithms cannot be implemented directly in these devices. This equipment is susceptible to hacking and DDoS attacks. Blockchain offers authentication and authorization, which reduces the likelihood of such attacks.
- Improved scalability: The IoT hardware needs to be installed on a large fleet of vehicles that generate an enormous amount of data every second, and the number of vehicles equipped with this system will continue to grow. Blockchain offers superior scalability compared to a centralized cloud server. Blockchain is fundamentally distinct from centralized systems and is designed to address issues such as scalability.
- Interoperability: Blockchain offers a uniform interface that is compatible with a variety of software and hardware environments. It improves the interoperability between IoT devices equipped with blockchain and other environments. In the current implementation of the system, both humans and other machines or software can access and utilize the data of any monitored vehicle driver. The data can be extracted, searched, and visualized by humans, machines, and software-based platforms.
- Execution time: A solitary organization operates and maintains a centralized system. If this is improperly loaded with unwanted software, consumers will be prevented from using it. This will have an impact on the execution times of all consumers. If the system is distributed, as opposed to centralized, then in the event that a node becomes severely overloaded or is destroyed, there will be a minimal performance change on the blockchain. This is because the blockchain operates on chains of blocks.

## 4. Results

**Figure 11:**
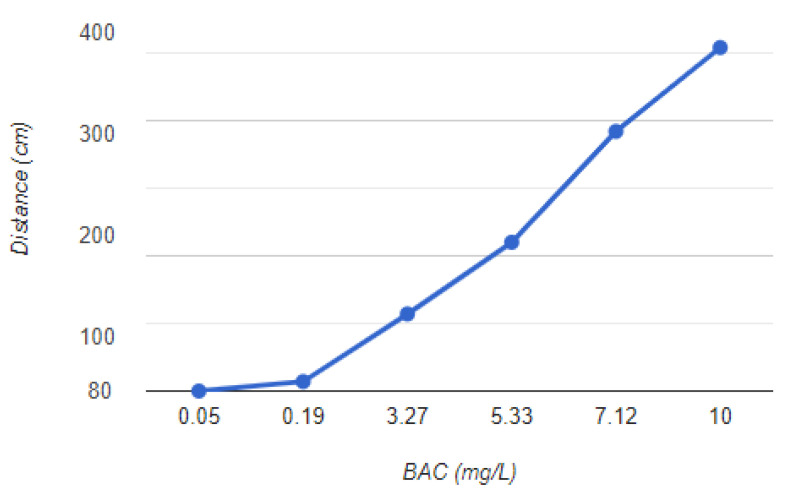
BAC vs. the distance from the driver’s mouth.

A methylated spirit that is 90% pure alcohol was used for testing the alcohol sensors and calculating BAC levels. This spirit is used in the industry. According to the MQ3 sensor’s datasheet, which can be found at [36], the sensor is capable of detecting BAC concentrations ranging from 0.05 to 10 mg/L. The maximum and minimum possible analog values for the sensor are set at 334 and 80, respectively. With these values, it is possible to calculate the BAC value for the sensor values that fall within this range. The relationship between the BAC in mg/L and the increase in the analog sensor value is shown in Table 4; Figure 11 shows the visual presentation of the relationship between distance and the BAC in mg/L.

### 4.1. Protection against Adversarial Attacks

The system protects against a variety of attacks and flaws in data security and is briefly discussed here.

#### 4.1.1. Data Mutability

**Figure 12:**
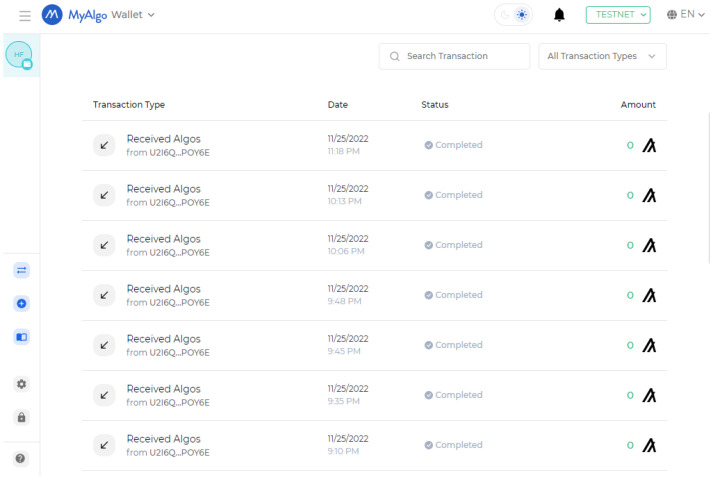
Drives read-only transactions to ensure the immutability of data.

A traditional traffic monitoring system with a centralized system and, therefore, a centralized database where all the data are stored, is susceptible to change. The data can be modified or even deleted at any time by anyone with sufficient access rights. This issue is resolved as a result of the application of blockchain technology. Figure 12 illustrates the blockchain transactions for the proposed approach. Once the transaction is completed, it becomes a permanent part of the system. None of the transactions may be removed or altered by an individual authority.

#### 4.1.2. Identity Theft

Identity theft is a type of cyberattack in which a hacker obtains and uses an individual’s personal information without their knowledge or consent, such as their name, address, and financial information. This information could be used by the hacker to impersonate the victim, commit fraud, or other crimes. Users’ identities can be verified securely and transparently using blockchain-based systems, making it more difficult for hackers to impersonate legitimate users. The system has the potential to aid in the prevention of identity theft by providing a secure and transparent method of verifying the identity of individual vehicle drivers.

#### 4.1.3. Man-in-the-Middle Attack

**Figure 13:**
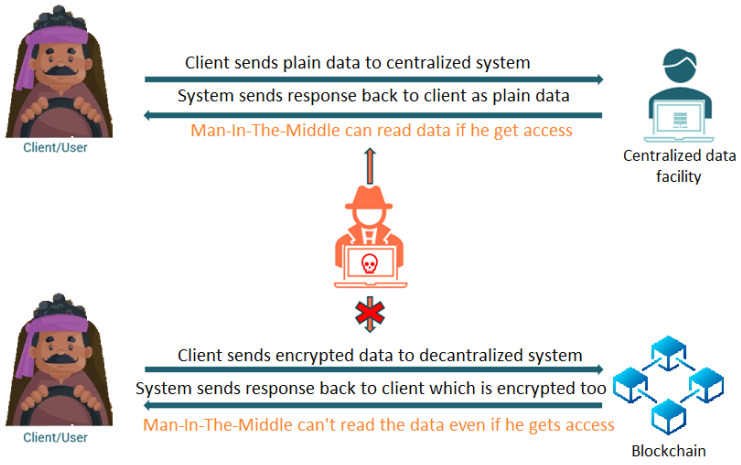
DrunkChain’s protection against man-in-the-middle attacks.

Blockchain technology enables secure communication between parties without requiring a central authority. This can aid in the prevention of man-in-the-middle attacks, in which a hacker intercepts and modifies communications between two parties. The data sent to the blockchain are not transmitted in their original formats, but rather in encrypted formats, ensuring their security. Figure 13 demonstrates how the attack is thwarted using a decentralized system, such as a blockchain.

**Figure 14:**
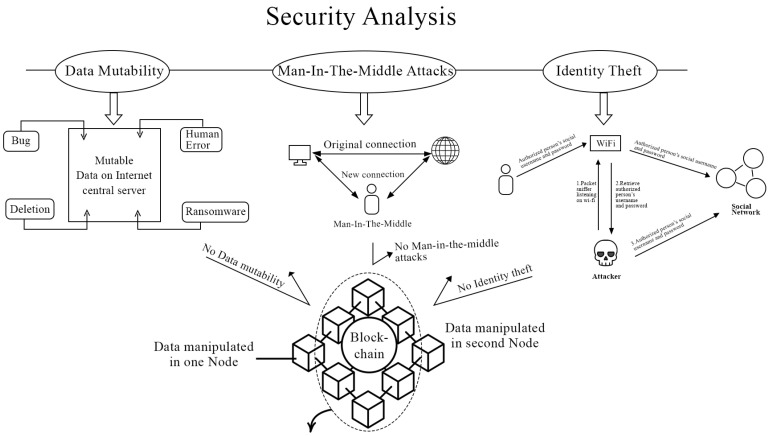
Security analysis of the DrunkChain approach.

Figure 14 illustrates the threats that can damage sensitive data, such as the live status of the driver if he is responsible for an accident. This stored data can be retrieved by authorized police stations, but if the data are altered by an unauthorized individual, the sensitive data may be altered or lost. Therefore, a robust and secure platform is required for storing driver statistics.

### 4.2. Comparison with Existing Systems

Table 5 provides a comparison of the current system to other systems that are already in place. When comparing different systems using the same criteria, there are five primary systems that are taken into account.

The first candidate for comparison is the traditional system, in which police officers manually stop and inspect each vehicle whose driver appears to be an alcohol-impaired suspect. This system is inefficient, slow, time-intensive, and resource-intensive. Police officials can manually identify drunk drivers by smelling their breath and observing how they drive. As humans are prone to making mistakes, there is a high likelihood that a drunk driver will be overlooked. A drunk driver can substitute another person for himself in the vehicle, and police can only check the driver before letting him go. The scalability of this system is also poor. Scaling requires additional human resources.

In [24], the authors proposed a straightforward system for detecting potholes and alcohol. This system lacks security and is extremely susceptible to attacks. There are no data transfers over the network and scalability can, therefore, be a big challenge. In [18], the authors proposed a mobile application that uses a phone’s built-in orientation sensor to determine whether or not a driver is intoxicated. This system is extremely ineffective because it lacks sensors to detect the amount of alcohol in the driver’s breath. Even if a driver is driving in poor road conditions, the system may declare him to be intoxicated. This system does not utilize cloud storage to store data and scalability is challenging.

Another system for comparison is the one proposed in [20], which incorporates blockchain implementation in the e-government sector. It illustrates how blockchain technology can be utilized for the storage and retrieval of public data. It primarily focuses on the security aspects and their implementations but makes no mention of alcohol detection and prevention. Furthermore, no hardware modules or sensors are considered for the prevention of accidents caused by drunk drivers. In [37], the authors presented a system that detects intoxicated driving based on the driver’s BAC and motion stability. In this work, the blockchain was implemented, but the authors did not specify the type of blockchain implemented or how it was integrated with hardware. Only the Arduino board is used as the central processing unit in this work, which is insufficient to implement blockchain technology.

Performance comparison results given in Table 5 indicate that the proposed system is superior to existing systems concerning BAC detection and energy efficiency. Moreover, the proposed system is attack-tolerant due to the implementation of blockchain technology. In addition, to accommodate more sensors and communication technology, the system is scalable for further extension.

### 4.3. Performance Tests

To ensure the connectivity of hardware and reliability of the system, the below-mentioned tests were performed on the system.

#### Network Connectivity Tests

**Figure 15:**
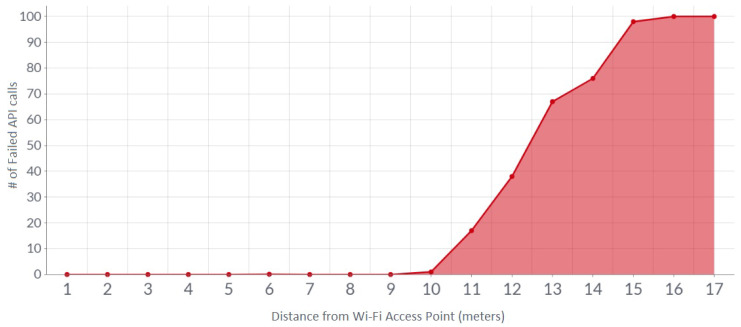
API success rate vs. distance from the Wi-Fi access point.

The built-in Wi-Fi module of Raspberry Pi could connect to a Wi-Fi network up to 20 m [38]. The internet source for the system was a 4G LTE-powered portable Wi-Fi hotspot and the device name was EVO Charji [39]. Raspberry Pi could connect to this internet device within the range of 17 m. The first test involved monitoring the system’s network health at a distance from the Wi-Fi source, which was the EVO Charji device. A total of 100 API calls were made to store data on the blockchain for each batch at different distances from the Wi-Fi source, as can be seen in Figure 15.

**Figure 16:**
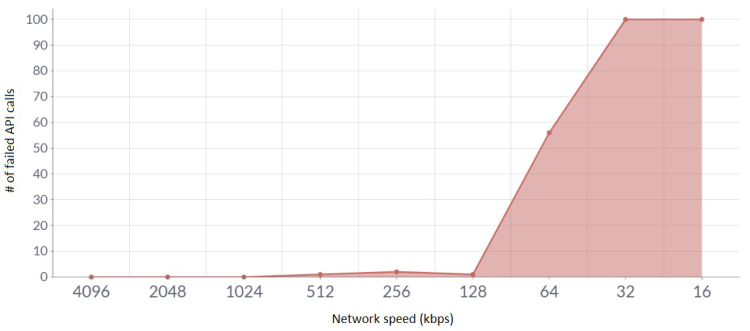
Internet speed vs. distance from the Wi-Fi access point.

The success rate of API calls was also tested based on internet speed. This test was performed by hitting batches of 100 APIs by manually controlling the internet speeds, which could be seen in Figure 16.

## 5. Limitations, Future Directions, and Practical Applications of Study

This paper proposes a method that employs blockchain technology and the Internet of Things (IoT) to combat drunk driving and reduce related traffic accidents. This section provides some basic constraints, future research directions, and potential applications for the suggested system, in addition to the study’s specifics.

### 5.1. Limitations of the Study

- Scalability: Implementing a blockchain-based IoT system to prevent drunk driving may result in scalability issues. As the Internet of Things (IoT) devices and system participant numbers grow, the blockchain network may encounter challenges with transaction processing speeds and data storage capacity.
- Privacy concerns: Collecting and disseminating sensitive information regarding alcohol intake and driving behavior can lead to privacy concerns. It is essential to protect the security and privacy of this information to prevent its misuse or unauthorized access.
- Adoption and compliance: The successful implementation of the proposed system is contingent on the participation and cooperation of many stakeholders, including drivers, vehicle manufacturers, law enforcement agencies, and regulatory authorities. Encouraging system adoption and enforcing system compliance can be challenging.

### 5.2. Future Research Directions

- Performance optimization: Further study can concentrate on improving the scalability, transaction processing speed, and energy efficiency of a blockchain-based Internet of Things system. Investigating different consensus techniques or designing hybrid designs may assist in enhancing the performance of the system.
- Privacy-preserving methods: Developing privacy-preserving procedures, such as zero-knowledge proof or safe multiparty computation, enables the sharing of relevant data without jeopardizing the privacy of people. Investigating improved cryptography algorithms can alleviate privacy concerns.
- Machine learning and predictive analytics: Integrating machine learning algorithms and predictive analytics can assist in identifying patterns and predicting possible drunk driving events. Improving the system’s ability to recognize harmful conduct in real time can result in more effective preventative measures. Further study might concentrate on improving the scalability, transaction processing speed, and energy efficiency of the blockchain-based Internet of Things system. Investigating different consensus techniques or designing hybrid designs may assist in enhancing the performance of the system.

### 5.3. Applications in Practice

- Prevention and awareness: The study’s findings can be utilized to develop educational and awareness efforts aimed at both drivers and the general public. Accidents can be reduced by promoting safe alcohol intake and emphasizing the necessity of preventing drunk driving.
- Law enforcement: The suggested technology can aid law enforcement agencies by delivering real-time information and notifications regarding possible drunk driving events. These data can assist them in prioritizing their resources and acting swiftly to avert mishaps.
- Insurance industry: Using the obtained results, insurance businesses might build unique goods and services, for instance, delivering premium discounts to individuals who actively participate in the blockchain-based IoT system and display safe driving behaviors.
- Manufacturers of motor automobiles: The findings can affect the design and development of vehicles to include improved sensors and technology that can identify indicators of alcohol impairment in drivers. This can contribute to the development of safer automobiles and lower the likelihood of drunk driving-related incidents.

## 6. Conclusions

This study presents a novel secure, scalable, and commercially feasible solution for drunk driving detection. The conventional procedure, where police officers personally stop and inspect each car whose driver appears to be under the influence of alcohol, is unnecessarily slow, time- and resource-consuming, and ineffective. In addition, human error and bias can lead to wrong identification or allow the culprit to leave. Although several automated systems exist, such systems are prone to adversarial attacks, are expensive, and lack scalability. The presented system incorporates IoT sensors, including BAC level identification, vehicle motion analysis, and GPS sensors, which provide accurate BAC estimation, the driver’s driving behavior, and vehicle location. In addition, the use of blockchain technology makes the system secure against cyberattacks. The records that are written are immutable, and the information transfer and retrieval are both very scalable and secure. The performance comparison with existing systems shows the superiority of the proposed systems in terms of security, scalability, and energy efficiency. In the future, we intend to extend this work by implementing machine learning models to analyze motion data for BAC identification. Video data of drivers may also be added with a BAC sensor to obtain more accurate results for driver intoxication.

## Figures and Tables

**Table 1:** Total number of victims in fatal automobile accidents.

Nature of Data	Count
Total fatalities in the country [4]	27,582 per year
Total accidents in Karachi city [5]	154 in 10 months
Total fatalities caused by inebriated drivers in Karachi city [5]	150 in 10 months

**Table 2:** Emphasis and omissions in each study that contributes to the formation of the safe driving system.

Reference	Focus of the Paper	Gaps				
		Authentication and Authorization	Data Encryption and Security	Backup and Recovery	Underlying Technology Used	Problem Solution
[7]	Measures to shut down and track vehicles to make the roads safer from drunk driving accidents	0	0	0	1	2
[8]	Implementation of an embedded system with an alcohol sensor, which enables the vehicle to prevent the drunk individual from driving	0	1	1	0	1
[9]	A system that controls traffic using IoT and AI by signaling and detecting the roads and traffic	1	0	1	1	2
[10]	A safe driving mechanism that involves tracking driving behavior with detection accuracy and alarm rates	0	0	0	1	1
[11]	Online system that detects anomalies by quantitatively evaluating the information of the driver	1	0	1	1	1
[12]	An IoV-based system that detects if a driver is fatigued via neural networks (ML) and the normalization algorithm	1	1	1	1	1
[13]	DL and AI-based systems to recognize driving hazards for light transport vehicles (LTVs), providing early warnings prior to predicted collisions	2	1	0	1	2
[14]	Identifies the factors that contribute to the overall driving experience and compares these factors between older and younger drivers	0	0	0	1	1
[15]	A virtual reality (VR) driving ’game’ that educates the public more effectively on the hazards of drunk driving using an evidence-based approach; includes real alcohol-impaired participants	1	2	1	1	1

**Table 3:** Summary of the variations of technological indicators across different studies.

Technological Dissection of Each System Presented in the Study	Study	Qty
Android	[18,19]	2
Modules using Wi-Fi	[7,9,11,17,18]	5
Arduino	[8,16]	2
Blockchain	[17,20,21]	3
Sensors	[7,8,22]	3
GPU	[7,8,10,12,23]	5
IoT	[7,9,16,19,20,22,23,24,25]	9
GPS	[7,8,10,16,22,24]	6
AI and machine learning models	[9,10,11,12,19,23,25]	7

**Table 4:** BAC content in relation to the driver’s mouth distance.

Distance (cm)	Sensor Value	BAC (mg/L)
0	334	10
10	272	7.12
20	190	5.33
30	137	3.27
40	87	0.19
50	80	0.05

**Table 5:** Evaluation of the proposed system concerning existing systems.

Evaluation Parameter	Traditional System	[24]	[18]	[20]	[37]	Proposed
Alcohol detection and prevention system	×	✓	×	×	✓	✓
Security	×	×	×	✓	✓	✓
Security implementation details are valid and well explained	×	×	×	✓	×	✓
Scalable	×	×	×	✓	✓	✓
Energy efficient	×	×	×	×	✓	✓

## Data Availability

Not applicable.

## References

[1] (2021). Estimated Worldwide Motor Vehicle Production from 2000 to 2021. https://www.statista.com/statistics/262747/worldwide-automobile-production-since-2000/.

[2] (2022). The Provincial Motor Vehicles Ordinance 1965. http://punjablaws.gov.pk/laws/189.html.

[3] (2018). Global Status Report on Alcohol and Health 2018. https://www.who.int/publications/i/item/9789241565639.

[4] (2018). Global Status Report on Road Safety. https://www.who.int/publications/i/item/9789241565684.

[5] (2020). Deaths by Inebriated Drivers In Pakistan. https://www.dawn.com/news/1598181.

[6] Sanghvi K. (2018). Drunk driving detection. Comput. Sci. Inf. Technol..

[7] Uzairue S., Ighalo J., Matthews V.O., Nwukor F., Popoola S.I. (2018). IoT-enabled alcohol detection system for road transportation safety in smart city. Proceedings of the International Conference on Computational Science and Its Applications.

[8] Gupta A., Ojha S., Kumar V., Singh V., Malav V. (2016). Alcohol detection with vehicle controlling. Int. J. Eng. Manag. Res. (IJEMR).

[9] Deb T. (2021). IoT based road traffic control system for Bangladesh. Int. J. Recent Technol. Eng..

[10] Chen L.W., Chen H.M. (2020). Driver behavior monitoring and warning with dangerous driving detection based on the internet of vehicles. IEEE Trans. Intell. Transp. Syst..

[11] Zhang M., Chen C., Wo T., Xie T., Bhuiyan M.Z.A., Lin X. (2017). SafeDrive: Online driving anomaly detection from large-scale vehicle data. IEEE Trans. Ind. Inform..

[12] Han M., Wu J., Bashir A.K., Yang W., Imran M., Nasser N. Adversarial learning-based bias mitigation for fatigue driving detection in fair-intelligent iov. Proceedings of the GLOBECOM 2020-2020 IEEE Global Communications Conference.

[13] Halim Z., Sulaiman M., Waqas M., Aydın D. (2022). Deep neural network-based identification of driving risk utilizing driver dependent vehicle driving features: A scheme for critical infrastructure protection. Ambient Intell. Humaniz. Comput..

[14] Park D.E., Park S.E. (2021). Factors affecting perceived safety and enjoyment based on driver experience. Transp. Res. Part F Traffic Psychol. Behav..

[15] Andrews J., Shareef Z., Mohammed M., Nwobi E., Masri-zada T., Head T., Zohr T., Head D., Commissaris R. (2021). A ‘Hands on’ Public Service Program to Help People Stay Sober and Safer on the Roadway. Sustainability.

[16] Šarac M., Pavlović N., Bacanin N., Al-Turjman F., Adamović S. (2021). Increasing privacy and security by integrating a Blockchain Secure Interface into an IoT Device Security Gateway Architecture. Energy Rep..

[17] Hjalmar D., Jamente C., Pillai Sivakumar I. (2018). How Blockchain and AI accelerate the application of IoT technology to keep drivers’ safe on the road. Proc. Stud.-Fac. Res. Day CSIS Pace Univ..

[18] Dai J., Teng J., Bai X., Shen Z., Xuan D. Mobile phone based drunk driving detection. Proceedings of the 2010 4th International Conference on Pervasive Computing Technologies for Healthcare.

[19] Ma C., Dai X., Zhu J., Liu N., Sun H., Liu M. (2017). Drivingsense: Dangerous driving behavior identification based on smartphone autocalibration. Mob. Inf. Syst..

[20] Kassen M. (2022). Blockchain and e-government innovation: Automation of public information processes. Inf. Syst..

[21] Qashlan A., Nanda P., He X., Mohanty M. (2021). Privacy-preserving mechanism in smart home using blockchain. IEEE Access.

[22] Ullah A., Sumiraj A., Shanawoaz M., Kabir A. (2020). IoT based vehicle speed monitoring and controlling system for reducing accident occurs on the road in Bangladesh. J. Adv. Parallel Comput..

[23] Biswal A.K., Singh D., Pattanayak B.K., Samanta D., Yang M.H. (2021). IoT-based smart alert system for drowsy driver detection. Wirel. Commun. Mob. Comput..

[24] Pingale A., Farheen T. (2021). Pothole and Alcohol Detection using IoT. Int. Res. J. Eng. Technol..

[25] Ramprasad M., Srinivas K. (2019). Tracking alcoholic driving using with artificial intelligence and IoT devices. Int. J. Innov. Technol. Explor. Eng..

[26] Altaf A., Abbas H., Iqbal F., Derhab A. (2019). Trust models of internet of smart things: A survey, open issues, and future directions. J. Netw. Comput. Appl..

[27] Altaf A., Abbas H., Iqbal F., Khan M.M.Z.M., Daneshmand M. (2021). Robust, Secure, and Adaptive Trust-Oriented Service Selection in IoT-Based Smart Buildings. IEEE Internet Things J..

[28] Altaf A., Abbas H., Iqbal F., Khan M.M.Z.M., Rauf A., Kanwal T. (2021). Mitigating service-oriented attacks using context-based trust for smart cities in IoT networks. J. Syst. Archit..

[29] Altaf A., Abbas H., Iqbal F., Khan F.A., Rubab S., Derhab A. (2021). Context-oriented trust computation model for industrial Internet of Things. Comput. Electr. Eng..

[30] Altaf A., Iqbal F., Latif R., Yakubu B.M., Latif S., Samiullah H. (2022). A Survey of Blockchain Technology: Architecture, Applied Domains, Platforms, and Security Threats. Soc. Sci. Comput. Rev..

[31] Fu Y., Li C., Yu F.R., Luan T.H., Zhang Y. (2022). A Survey of Driving Safety With Sensing, Vehicular Communications, and Artificial Intelligence-Based Collision Avoidance. IEEE Trans. Intell. Transp. Syst..

[32] (2022). I2C Bus. https://en.wikipedia.org/wiki/I%C2%B2C.

[33] (2022). Adafruit MPU6050 Library. https://github.com/adafruit/Adafruit_MPU6050.

[34] (2022). Ubuntu. https://ubuntu.com/.

[35] (2022). Algorand Blockchain. https://www.algorand.com/.

[36] (2022). MQ3 Sensor Datasheet. https://www.sparkfun.com/datasheets/Sensors/MQ-3.pdf.

[37] Soner S., Litoriya R., Pandey P. (2022). Integrating Blockchain Technology with IoT and ML to Avoid Road Accidents Caused by Drunk Driving. Wirel. Pers. Commun..

[38] (2023). Purestake API. https://notenoughtech.com/raspberry-pi/raspberry-pi-internet-speed/.

[39] (2023). Purestake API. https://charji.ptcl.com.pk/.

